# Economic evaluation of weekends-off antiretroviral therapy for young people in 11 countries

**DOI:** 10.1097/MD.0000000000009698

**Published:** 2018-02-02

**Authors:** Luis Enrique Tierrablanca, Jessica Ochalek, Deborah Ford, Ab Babiker, Diana Gibb, Karina Butler, Anna Turkova, Susan Griffin, Paul Revill

**Affiliations:** a Tecnología e Información para la Salud, Mexico City, Mexico; b Centre for Health Economics, University of York, York; c Medical Research Council Clinical Trials Unit, University College London, London; d Our Lady's Children's Hospital, Dublin; e Great Ormond Street Hospital, London, UK.

**Keywords:** antiretroviral therapy, cost–benefit analysis, HIV, young people

## Abstract

**Objectives:**

To analyze the cost effectiveness of short-cycle therapy (SCT), where patients take antiretroviral (ARV) drugs 5 consecutive days a week and have 2 days off, as an alternative to continuous ARV therapy for young people infected with human immunodeficiency virus (HIV) and taking efavirenz-based first-line ARV drugs.

**Methods:**

We conduct a hierarchical cost-effectiveness analysis based on data on clinical outcomes and resource use from the BREATHER trial. BREATHER is a randomized trial investigating the effectiveness of SCT and continuous therapy in 199 participants aged 8 to 24 years and taking efavirenz-based first-line ARV drugs in 11 countries worldwide. Alongside nationally representative unit costs/prices, these data were used to estimate costs and quality adjusted life years (QALYs). An incremental cost-effectiveness comparison was performed using a multilevel bivariate regression approach for total costs and QALYs. Further analyses explored cost-effectiveness in low- and middle-income countries with access to low-cost generic ARV drugs and high-income countries purchasing branded ARV drugs, respectively.

**Results:**

At 48 weeks, SCT offered significant total cost savings over continuous therapy of US dollar (USD) 41 per patient in countries using generic drugs and USD 4346 per patient in countries using branded ARV drugs, while accruing nonsignificant total health benefits of 0.008 and 0.009 QALYs, respectively. Cost-effectiveness estimates were similar across settings with access to generic ARV drugs but showed significant variation among high-income countries where branded ARV drugs are purchased.

**Conclusion:**

SCT is a cost-effective treatment alternative to continuous therapy for young people infected with HIV in countries where viral load monitoring is available.

## Abstract 2

Supplemental Digital Content is available in the text

## 1 Introduction

Thirty years after acquired immunodeficiency syndrome (AIDS) was first recognized, considerable progress has been made in combating the epidemic. The public health landscape was transformed with the emergence of effective human immunodeficiency virus-1 (HIV-1) therapies and the subsequent global expansion of access to these treatments.^[1,2]^ Evidence has shown that people who have access to antiretroviral (ARV) drugs early in the course of infection may live a near-normal lifespan.^[3]^

Despite these achievements, challenges still exist for vulnerable groups, such as young people who are more likely to drop out of care and have lower viral suppression and adherence rates than adults.^[4–7]^ The 2016 World Health Organization HIV Treatment Guidelines called for adolescent friendly treatment guidelines, yet the evidence on approaches to achieve this remains limited.^[8,9]^

One option that offers promise is short-cycle therapy (SCT), in which patients have weekends off from taking long-acting ARV drugs. This was shown to be virologically noninferior to continuous treatment in the BREATHER trial, which assessed young people, as well as among adults in small adult trials.^[10–13]^ Findings from a qualitative study using a subsample of BREATHER showed that participants described a positive SCT experience and a preference to SCT over continuous therapy.^[14]^

As yet no information exists to guide policymakers about the value for money of SCT compared with continuous therapy for HIV-positive young people. This study investigates the cost-effectiveness of SCT in the 11 countries that took part in the BREATHER trial and explores if the economic results could be applicable to other settings.

## 2 Methods

The cost-effectiveness analysis compared SCT and continuous therapy using individual patient-level data from BREATHER on resource use and quality adjusted life years (QALYs) over a 48-week time horizon. Participants were aged 8 to 24 years, and must have been stable on first-line efavirenz with 2 nucleoside reverse transcriptase inhibitors with HIV-1 ribonucleic acid viral load <50 copies/mL for 12 months or longer. The trial protocol was approved by the ethics committees in participating centers in Europe, Africa, and the United States. Parents or guardians and older participants provided written consent; young children gave assent appropriate for age and knowledge of HIV status, as per guidelines for each participating country. The trial is described in detail elsewhere.^[10]^ Participants were randomized 2 to 4 weeks after screening and then assessed clinically, including viral load and T lymphocytes measurements, at weeks 4 and 12, and then every 12 weeks for a total of 48 weeks’ follow-up.

Due to heterogeneity in ART prices across countries, the trial sample was divided into 2 groups: countries that access generic drugs through the Global Fund for AIDS, tuberculosis, and malaria procurement systems (“generic”: Thailand, Uganda, and Ukraine); those who pay for brand name ARV drugs (“branded”: Argentina, Belgium, Denmark, Germany, Ireland, Spain, the United Kingdom, and the United States).

Resource use data is taken from the BREATHER trial from case report forms using a healthcare provider perspective, which includes only direct medical costs. Unit prices for the generic medications were extracted from the Global Fund and Médecins Sans Frontières.^[15,16]^ For the high-income countries, the ARV drug costs were obtained from local sources.^[17–24]^ The costs of inpatient care were obtained from the WHO-CHOICE dataset, while test costs were obtained from other studies.^[25–30]^ Monetary values are presented in 2015 US dollar (USD) .

Quality of life (QoL) was measured in the trial using the Pediatric Quality of Life (PedsQL) tool at randomization (0 weeks), 24 and 48 weeks. Although children-specific and widely validated, PedsQL is a nonpreference-based measure so cannot directly be used to calculate QALYs.^[31,32]^ To obtain QALYs, the PedsQL responses were mapped onto the EQ-5D health status descriptive tool using results from a previous exercise conducted in the United Kingdom (UK).^[33]^

A bivariate model specification was used to model costs and health outcomes simultaneously.^[34]^ The use of a multilevel specification was assessed as the trial data were hierarchical with patients nested into sites nested into countries (presenting a 3-level structure). Due to the objective of generating evidence for any country considering SCT, the appropriate hierarchical estimation method was the random-effects specification rather than a fixed-effects approach.^[35]^ Potential patient-level covariates were identified from the information collected in BREATHER.

Results are reported as incremental net monetary benefit (difference in outcomes multiplied by the cost-effectiveness threshold, less difference in costs). Two types of results were obtained: “pooled” (i.e., branded and generic groups) and country-specific (analyses by country). Country-specific values were estimated through empirical Bayes predictions (shrinkage estimators) using the random-coefficients specification.^[35]^ The cost-effectiveness thresholds were drawn from Woods et al.^[36]^ Positive incremental NMB indicates an intervention is cost-effective.

Values were missing for ART doses (12.1%), ART intake frequency (number of pills taken/day, 16.2%)^1^, and PedsQL measure for weeks 0, 24, and 48 at 19.6%, 17.6%, and 16.6%, respectively. Unit costs of laboratory tests were missing for 4 countries: Denmark, Spain, Germany, and Belgium.

As doses and intake frequency were similar between patients within the same cluster, ART cost data were imputed at the resource use level, using the country-specific mode. Where the cost of laboratory tests was not available, the highest unit cost in the generic/branded drug group to which the country belongs was used.

On the health benefit side of the trial, a descriptive analysis of missing data was performed in order to select the best method for handling the missing values (see Supplementary Material: eMethods for further details). According to this analysis, the data were nonmonotone missing at random with multiple follow-ups. Therefore, the best technique for imputing missing values is multiple imputation (MI).^[37]^ To consider the hierarchical structure of the data, a 2-level structure in the imputation process was made using the software Realcom. The missing utility values were predicted in terms of gender, age group, and total cost at 6 months. The MI process was validated by comparing the distributions of the observed with the imputed data sets.

## 3 Results

### 3.1 Quality of life and costs

For the generic and branded groups, there was no significant difference between SCT and continuous therapy for PedsQL scores at week 0, 12, and 24, and total QALYs. Significant differences were identified for ARV drug and total costs in both groups (Table 1).

**Table 1:**
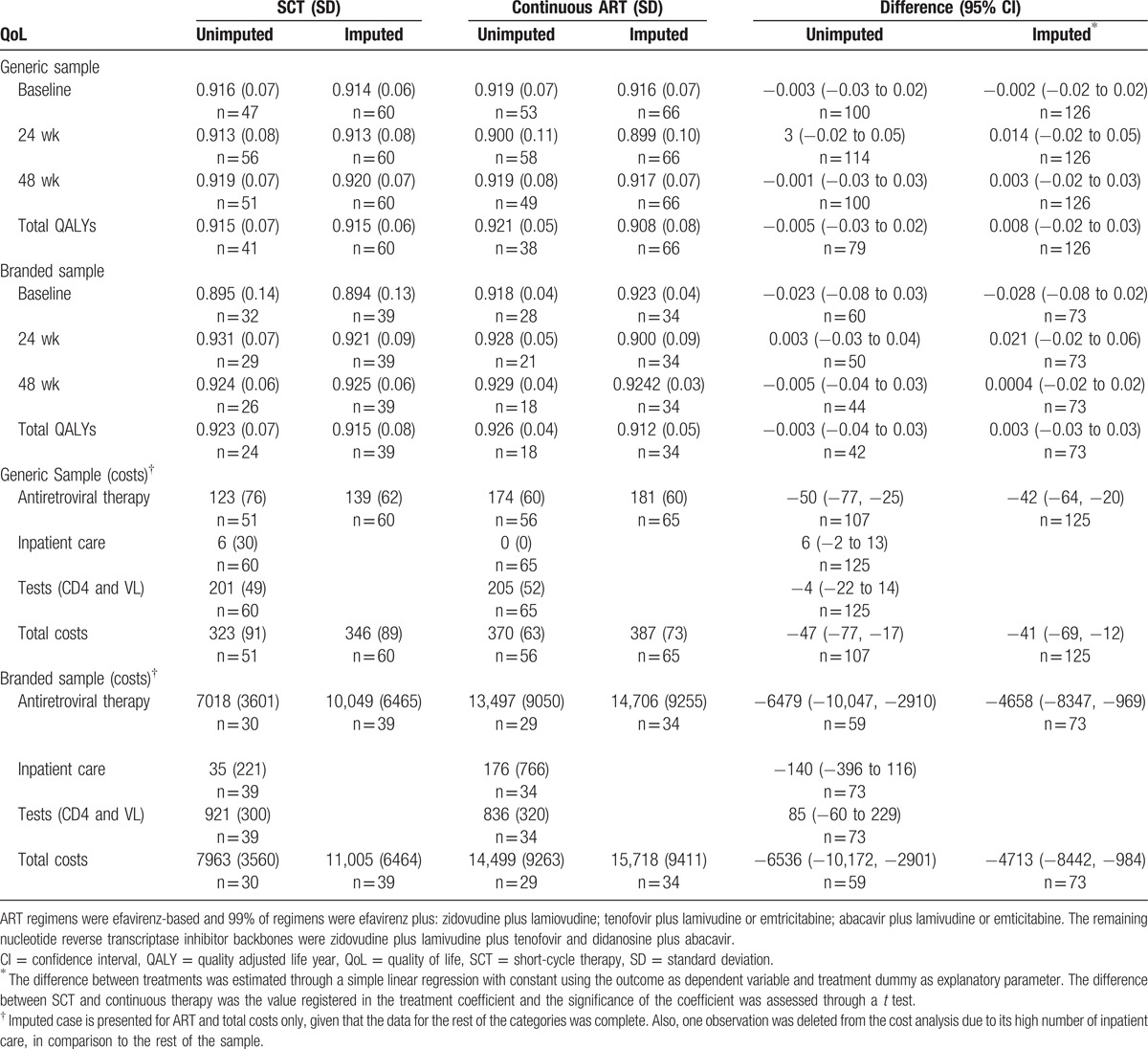
Pooled unimputed and imputed QoL and costs by trial arm.

**Figure 1:**
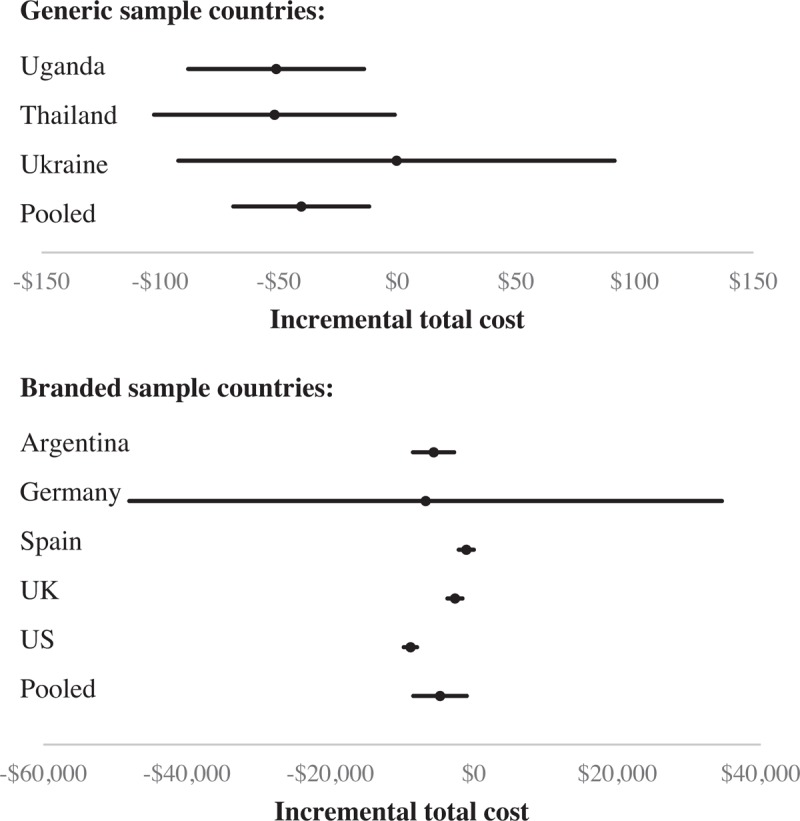
Incremental total cost of SCT compared with continuous therapy. A forest plot for incremental total cost of SCT versus continuous therapy is shown by country based on raw imputed data. Mean incremental costs (95% confidence intervals) are denoted by black circles (black lines). The pooled results include the values of all the patients inside a sample (generic/branded). SCT = short-cycle therapy.

At the country level, SCT significantly reduced total costs in most countries. In Germany and Ukraine, a decrease in total costs was nonsignificant (due to a very small sample size of 3 observations in Germany and an outlier in the SCT group in Ukraine who was treated with abacavir) (see Fig. 1).

### 3.2 Cost-effectiveness

For both groups, 3 modeling strategies were considered plausible. Differences between strategies result from different statistical approaches to the hierarchical data structure and the most appropriate strategy for each sample was determined by goodness-of-fit measures.

For the generic group, results show that countries differ minimally in measured QALYs and costs, and so a nonhierarchical specification is preferred. By contrast, for the branded group, estimation from the random-coefficients specification performs better than other strategies, implying that the pooled results may not apply to certain countries due to fundamental differences between clusters. See Supplementary Material: eMethods for the complete selection process.

The pooled results (Table 2) indicate that SCT offers significant total cost savings of USD 41 per patient over continuous therapy over the 48-week time horizon in countries using generic drugs and USD 4346 per patient in countries using branded ARV drugs while accruing nonsignificant QoL benefits of 0.008 and 0.009 QALYs, for the generic and branded groups, respectively. Country-specific results for both groups are reported in Table 3. Although pooled results differ from country-specific estimates in some cases, whether using pooled or country-specific results, SCT is a cost-effective alternative to continuous therapy in every country.

**Table 2:**
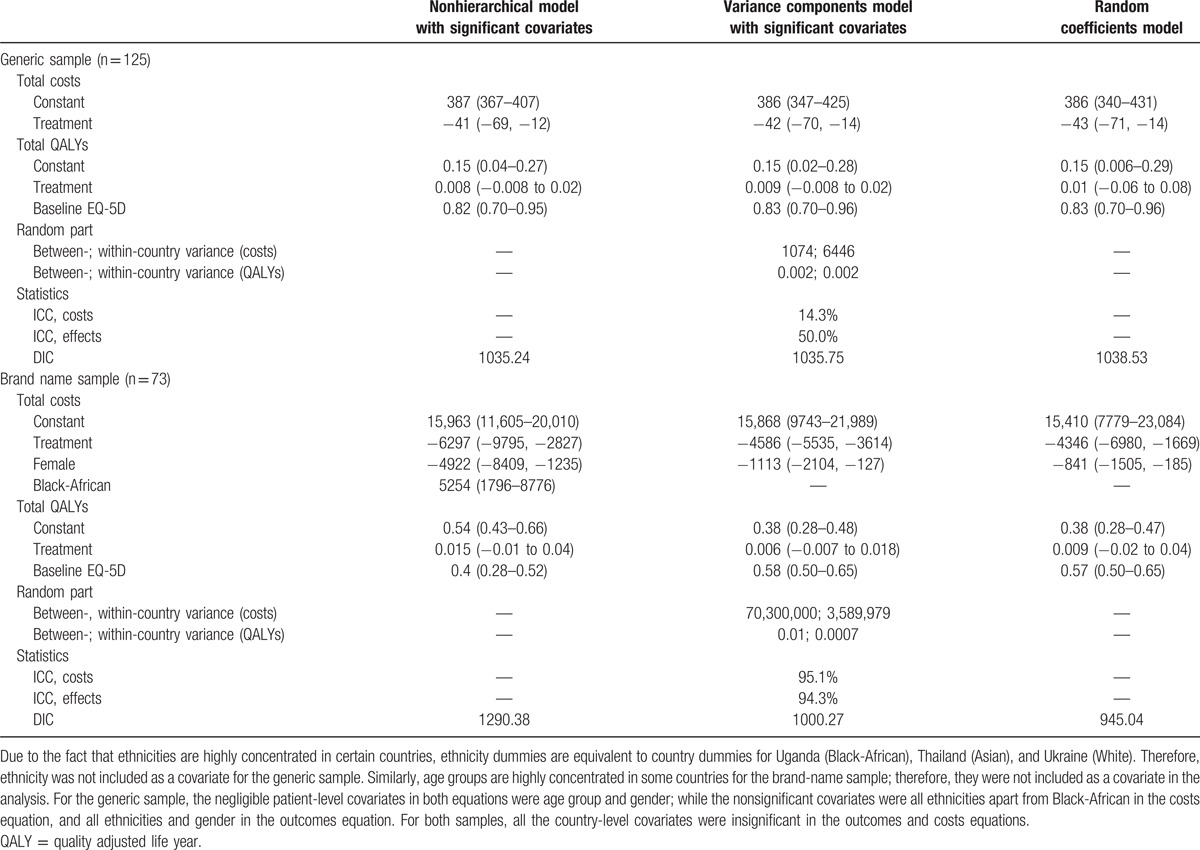
Model selection: Trial-wide results for both samples.

**Table 3:**
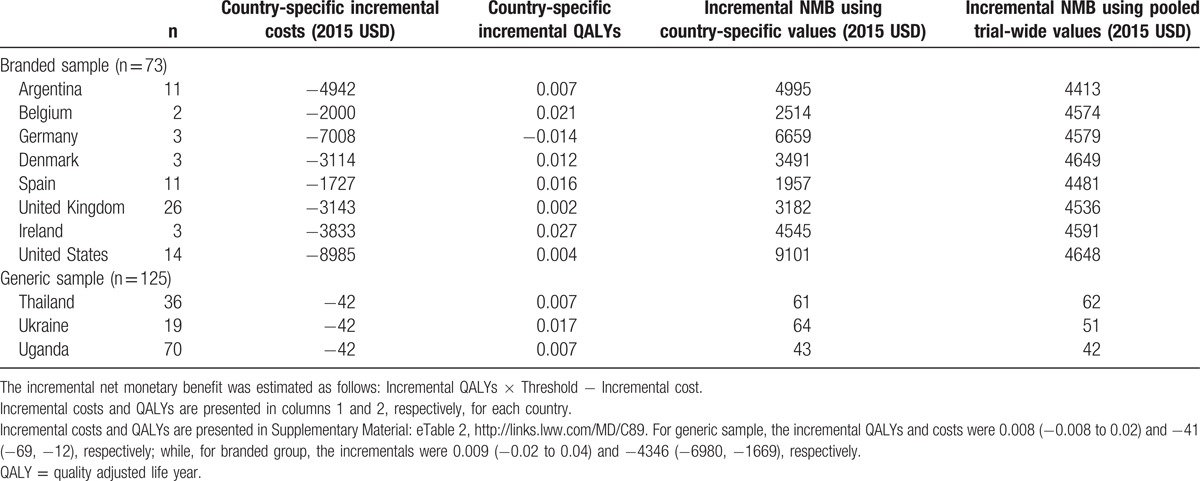
Cost effectiveness by group and country.

## 4 Discussion

The BREATHER multicountry trial showed that for HIV-infected young people, SCT with long-acting drugs was noninferior and maintained virological suppression compared with continuous therapy.^[10]^ Study participants expected SCT to be easier than staying on continuous therapy (88% at trial baseline), and at the end of the trial this expectation was confirmed with 90% of those in the SCT group reporting that SCT made life easier (than continuous therapy) particularly as going out with friends was easier.^[10]^ These findings were confirmed in a qualitative study using a subsample of BREATHER showed that participants described a positive SCT experience and a preference to SCT over continuous therapy.^[14]^ This study assesses the cost-effectiveness of SCT as an option for young people in a wide range of countries. We find SCT offers significant cost savings and small, nonsignificant gains in health-related QoL compared with continuous therapy in all countries.

The magnitude of cost-savings with SCT and resulting cost-effectiveness estimates depend, however, upon whether a country has access to generic ARV drugs or faces the full costs of branded drugs. Countries inside the Global Fund procurement program show substantial homogeneity in outcomes and costs indicating results could generalize to other low- and middle-income countries where viral load monitoring is available. Although there is more heterogeneity across the countries purchasing branded drugs, SCT is cost-effective in all the countries evaluated.

During the model selection process, statistical tests demonstrated that a multilevel approach was required for the branded sample; however, for the group of countries purchasing generic drugs, a simpler cluster analysis performed well. Given that SCT is highly likely to be cost-effective in all cases, other LMICs acquiring ARV drugs through the Global Fund can reasonably rely on the pooled results, although countries purchasing branded ARV drugs may wish to undertake cost-effectiveness studies of SCT specific to their own jurisdiction.

This finding implies that where baseline and relative risks are similar across settings and where countries have access to commonly procured commodities, such as through Global Fund mechanism, it is unlikely to be necessary to repeat cost-effectiveness analyses in all jurisdictions. However, where countries negotiate their own prices with manufacturers, jurisdiction-specific analyses may be preferable.

This study is the first to assess the cost-effectiveness of SCT, and is one of few that explores the economics of youth-friendly forms of HIV treatment. Adolescents are highlighted as a particularly vulnerable population in HIV epidemics and it is recognized that existing evidence on youth-friendly approaches is limited and of generally poor quality. Young people are expected to have many years of taking ARV drugs ahead of them and the option of SCT has the potential to effectively reduce treatment fatigue and improve clinical results over the longer term.

This study applies a distinctive methodology by implementing a multilevel framework in all steps of the analysis. Despite employing robust methods, the evaluation has some limitations. First, analysis was restricted to 48 weeks as QOL and cost data were only collected up to 48 weeks (time point for the trial's primary analysis). Since HIV patients may live a near-normal lifespan with treatment; a 48-week time horizon is limited and represents a truncated time-horizon. Subsequent follow-up to 144 weeks of the BREATHER trial participants, maintaining original randomization, demonstrated that noninferior virological suppression on SCT versus continuous therapy was sustained; there were also no significant differences in grade 3/4 adverse events or ART-related adverse events between groups.^[38]^ By 144 weeks 27/99 SCT participants had returned to continuous therapy (14 for viral rebound and 13 for other reasons; e.g., discontinuation of efavirenz, patient preference); most patients with viral rebound resuppressed on the same ART regimen. Although, the cost savings per year due to reduced ARV drug consumption on SCT are likely to diminish somewhat over time as some patients return to CT, BREATHER results suggest that SCT could offer substantial savings with ∼70% of SCT participants still taking weekends off out to 144 weeks.

Second, monitoring and clinic visits were more frequent and comprehensive than existing clinical practice in many countries. Where generic drugs are purchased the monitoring strategy is unlikely to include 3-monthly viral load monitoring as occurred in the trial. Further research may be warranted to assess if noninferiority and cost-effectiveness are maintained in real clinical settings even without enhanced monitoring.

Third, in some countries inside the branded sample, certain ARV drugs (i.e., efavirenz, tenofovir) will come off patent in the short run, which could have implications in the present analysis such as reducing the total cost gap between continuous therapy and SCT due to a considerable decrease in ARV drug acquisition costs.

A 4th potential limitation is that the health-related QoL mapping and resulting QALY estimates were based upon UK data and it is unclear whether there may be differences in values in other countries. However, there is no reason to believe use of UK health values in any way biased results one direction or another.

## 5 Conclusion

SCT, in which patients have weekends off from taking long-acting ARV drugs, is a cost-effective alternative to continuous therapy for young people. The cost effectiveness of SCT compared with continuous therapy was driven by lower ARV drug costs and differences in the other cost categories were negligible. Despite differences between countries, country-specific results reinforced the results of the pooled analysis; SCT did not have a significant impact on QoL but significantly reduced treatment costs.

Although countries differed in whether they had access to generic ARV drugs or purchased branded drugs, this study shows that SCT is cost-effective in all settings. As such, SCT can be considered as an adolescent-friendly alternative ART approach to current standard of care for young people.

## Acknowledgments

The authors thank to Andrew Phillips for comments. The authors thank all of the young people, their families, and staff from the centers participating in the BREATHER trial.

## Supplementary Material

Supplemental Digital Content

## Footnotes

1 Excluding missing data for existing treatment entries.
