# Economic evaluation of weekends-off antiretroviral therapy for young people in 11 countries: Erratum

**DOI:** 10.1097/MD.0000000000010012

**Published:** 2018-02-16

In article, “Economic evaluation of weekends-off antiretroviral therapy for young people in 11 countries”^[1]^, which appeared in Volume 97, Issue 5 of *Medicine*, the authors would like to add some details to the acknowledgement section. The acknowledgement should appear as:

We thank all of the young people, their families, and staff from the centres participating in the BREATHER trial (Supplementary material, Study contributors).
