# Economic evaluation of weekends-off antiretroviral therapy for young people in 11 countries: Erratum

**DOI:** 10.1097/MD.0000000000010158

**Published:** 2018-03-09

## Abstract

Supplemental Digital Content is available in the text

In article, “Economic evaluation of weekends-off antiretroviral therapy for young people in 11 countries”,^[1]^ which appeared in Volume 97, Issue 7 of *Medicine*, the authors would like to add some details to the acknowledgement section. The acknowledgement is missing the supplemental digital content link.

## Supplementary Material

Supplemental Digital Content

## References

[1] Tierrablanca LE Ochalek J Ford D Economic evaluation of weekends-off antiretroviral therapy for young people in 11 countries: Erratum. *Medicine*. 2018 97:e0012. 29384848 10.1097/MD.0000000000009698 PMC5805420

