# Supplementary material for: Economic evaluation of weekends-off antiretroviral therapy for young people in 11 countries
Source: Medicine (Baltimore). 2018 Feb 2;97(5):e9698. doi: 10.1097/MD.0000000000009698 (PMC5805420; doi:10.1097/MD.0000000000009698)
Supplement: Supplemental Digital Content [file medi-97-e9698-s001.docx]

**Online Only Supplemental Material**

eMethods: Imputation and Model selection.

**eMethods: Imputation and Model selection**

Imputationof QoL –related variables:

Mean imputation is not valid for missing outcomes because it underestimates uncertainty by not accounting for imputed values being estimated rather than known[1]. A descriptive analysis of missing data was undertaken to inform on the best method for handling the missing values in the health benefit side of the trial. The amount of missing data was analysed by trial group and follow-up assessment (Table 1).

**Table 1. Number and proportion of individuals with missing data by intervention.**

| Missing at week | SCT (n=99) | Continuous ART (n=100) |
| --- | --- | --- |
| 0 | 18 (24%) | 17 (22%) |
| 24 | 7 (9%) | 16 (21%) |
| 48 | 15 (20%) | 26 (33%) |

The proportion of individuals with missing data did not present a clear pattern in either trial arm and, after week 0, the amount of missing values was considerably higher for continuous ART. Both results indicated that the mechanism was unlikely to be missing completely at random (MCAR). The pattern of missing data was also analysed(Table 2).

**Table 2. Pattern of missing data.**

|  | Pattern | | |
| --- | --- | --- | --- |
| Frequency | Baseline utility | Utility at week 24 | Utility at week 48 |
| 89 | 1 | 1 | 1 |
| 18 | 1 | 0 | 1 |
| 13 | 0 | 1 | 0 |
| 13 | 1 | 1 | 0 |
| 9 | 1 | 0 | 0 |
| 6 | 0 | 0 | 0 |
| 2 | 0 | 0 | 1 |
| 2 | 0 | 1 | 1 |
| Total: 152 |  |  |  |

As previously identified, missing data was non-monotonic as individuals missing at onefollow-up could return to the trial in future follow-ups. Given the uneven patterns, the missing data was modelled in individual components of the total QALYs (by week) instead of directly imputing total QALYs.

The association between the missingness and baseline covariates was also analysed. For utility at 48 weeks, the missing values were related to the treatment. This information supported the assumption of covariate dependent missing at random(CD-MAR) or missing at random(MAR)[1]. An association between missingness and previously observed outcomes was also analysed, and showed that for utility at 24 weeks, the total cost at 6 months was related with the missing values. The significant association indicated that our data was likely MAR[1]. The logistic regression results for these analyses are given in Table 3-4.

**Table 3. Logistic regression results for missingness in terms of baseline covariates.**

|  | Odds ratio in logistic regression (95% Confidence Intervals) | | |
| --- | --- | --- | --- |
| Response Variable | **Missing data**  **at week 0** | **Missing data**  **at week 24** | **Missing data**  **at week 48** |
| Age group (13-18 years) | 2.18  (0.98-4.85) | 1.23  (0.40-3.77) | 0.61  (0.23-1.65) |
| Age group (19-25 years) | 7.25  (0.88-59.60) | 1  (omitted) | 1.11  (0.20- 6.24) |
| Treatment indicator | 0.80  (0.37-1.74) | 2.80  (0.82-9.53) | 3.11  (1.17- 8.22) |
| Female | 1.24  (0.57-2.71) | 1.21  (0.40-3.69) | 0.65  (0.026-1.63) |
| Baseline EQ-5D | Not included | 0.04  (0-1097.8) | 6.29  (0.01-3861.22) |
| N | 152 | 103 | 117 |

**Table 4. Logistic regression results for missingness in terms of previous outcomes.**

|  | Odds ratio in logistic regression (95% Confidence Intervals) | |
| --- | --- | --- |
| Response Variable | **Missing data**  **at week 24** | **Missing data**  **at week 48** |
| Baseline utility | 0.005  (0-336.80) | 0.82  (0-16463.31) |
| Utility at week 24 | Not included | 58.68  (0.08-44695.42) |
| Total costs at 6 months | .9995  (.9992-.9998) | 0.9995  (0.996-1.003) |
| Total costs at one year | Not included | 1.000602  (.998 -1.004) |
| N | 117 | 102 |

Under the non-monotonic MAR assumption with multiple follow-ups, the optimal technique for imputing missing values is Multiple Imputation (MI)[1] where each missing value is replaced with a set of predictions obtained using all the covariates that may be associated with the missingness[2]. This method effectively recognizes the uncertainty associated with the missing values and the estimated parameters in the imputation model[1]. Although MI allows for the efficient estimation of missing values, the existence of hierarchical structure must not only be reflected in the main model, but also in the multiple imputation process. In order to be able to reflect the two-level structure of the data the software Realcom was used [3]. To ensure enough degrees of freedom for the imputation regression, a two-level (patient and site) random effects model together with country fixed effects was used enabling country-level heterogeneity to be controlled for despite the small sample size.

All missing utility values were predicted in terms of gender, age group and total cost at 6 months. Baseline utility was not included as it is one of the response variables in the multivariate imputation model. The regressions are expressed as:

$${Baseline QALY}_{ij}=\beta_{0,1}+\beta_{1,1}t_{ij}+\sum_{j=3,1}^{8,1} \beta_{j}u_{ij}+e_{0,1j}+\epsilon_{0,1ij}$$

$${QALY at 24 weeks}_{ij}=\beta_{0,2}+\beta_{1,2}t_{ij}+\sum_{j=3,2}^{8,2} \beta_{j}u_{ij}+e_{0,2j}+\epsilon_{0,2ij}$$

$${QALY at 48 weeks}_{ij}=\beta_{0,3}+\beta_{1,3}t_{ij}+\sum_{j=3,3}^{8,3} \beta_{j}u_{ij}+e_{0,3j}+\epsilon_{0,3ij}$$

$$\left( \begin{matrix} \epsilon_{0,1ij} \\ \epsilon_{0,2ij} \\ \epsilon_{0,3ij} \end{matrix} \right)\sim N(0,\Omega_{\epsilon})$$

$$\left( \begin{matrix} e_{0,1ij} \\ e_{0,2j} \\ e_{0,3j} \end{matrix} \right)\sim N(0,\Omega_{e})$$

, where $u_{ij}$ includes the patient-level covariates, together with the country dummies.

30 imputed datasets were generated, and the results were combined using Rubin’s rule to generate an overall estimate and standard error [1].

Validation of MI:

As shown in Figure 1, the distributions of the observed data were similar to those estimated for the imputed data sets, suggesting that the imputation mechanism properly reflects the original data. Due to the large number of imputation series’ only a sample of the datasets are presented. The main difference between the distributions was the existence of some values over 1 for some imputed datasets. This was observed due to the inability to specify a truncated regression in Realcom, which could limit the values of EQ-5D between 0 and 1.

**Figure 1. Multiple imputation graphic validation.**

Model selection

The trial data exhibit a three-level structure, with patients nested into sites nested into countries, i.e. hierarchical data. Failure to acknowledge such clustering risks misleading conclusions in terms of the generalisability of the results[4]–[6].

A stepwise procedure to identify the model best representing the data was implemented. First, a non-hierarchical model was estimated and used to select statistically significant patient level covariates. Then, a two-level random intercepts specification was introduced. Despite the three-level structure of the data, given the limited amount of data and the risk of non-convergence in the relevant parameters, only two-levels of interest were specified: country and patients. If found necessary, country-specific covariates would be included to explain the between-country heterogeneity. Furthermore, given interest in country-specific estimates, the complexity of the hierarchical model was further increased by estimating a two-level random coefficients model with patient and country level covariates.

**References**

[1] R. Faria, M. Gomes, D. Epstein, and I. R. White, “A Guide to Handling Missing Data in Cost-Effectiveness Analysis Conducted Within Randomised Controlled Trials,” *Pharmacoeconomics*, vol. 32, no. 12, pp. 1157–1170, 2014.

[2] A. Burton, L. Jane, and S. Bryan, “Cost-effectiveness in clinical trials : using multiple imputation to deal with incomplete cost data,” *Clin Trials*, vol. 4, no. 2, pp. 154–161, 2007.

[3] J. J. R. Carpenter, H. Goldstein, and M. G. M. Kenward, “REALCOM-IMPUTE Software for Multilevel Multiple Imputation with Mixed Response Types,” *J. Stat. Softw.*, vol. 45, no. 5, pp. 1–12, 2011.

[4] R. Grieve, R. Nixon, S. Thompson, and J. Cairns, “J. Multilevel models for estimating incremental net benefits in multinational studies,” *Health Econ.*, vol. 16, no. December 2006, pp. 815–826, 2007.

[5] M. Hernandez and A. J. Wailoo, “Multilevel modelling of cost data: an aplication to thrombolysis and primary angioplasty in the UK NHS,” *HEDS*, 2010.

[6] A. Manca, P. C. Lambert, M. Sculpher, and N. Rice, “Cost-effectiveness analysis using data from multinational trials: the use of bivariate hierarchical modeling.,” *Med. Decis. Making*, vol. 27, no. 4, pp. 471–490, 2009.

# x

x
